# Chemical termination and interfacial redox behavior of freestanding SrTiO_3_

**DOI:** 10.1038/s41598-025-15902-z

**Published:** 2025-10-09

**Authors:** M. A. Wohlgemuth, K. Nayak, A. Kaus, L. Heymann, L.-K. Huang, A. Sarantopoulos, J. D. Thomsen, R. E. Dunin-Borkowski, V. Rouco, J. Santamaría, R. Dittmann, F. Gunkel

**Affiliations:** 1 Peter Grünberg Institute 7, Forschungszentrum Jülich GmbH, 52425 Jülich, Germany; 2 Jülich-Aachen Research Alliance (JARA-FIT), RWTH Aachen University, 52056 Aachen, Germany; 3 Ernst Ruska-Centre for Microscopy and Spectroscopy with Electrons, Forschungszentrum Jülich GmbH, 52428 Jülich, Germany; 4 GFMC, Dpto. Fisica de Materiales, Universidad Complutense de Madrid, 28040 Madrid, Spain

**Keywords:** Materials science, Nanoscience and technology

## Abstract

Tailoring oxide heterointerfaces has sparked the search for electronic and ionic phenomena in low-dimensional, confined systems. The fabrication of freestanding oxide membranes has further expanded the possible fields of application. Based on the structural vulnerability and physical confinement of such membranes, it remains a great challenge to achieve atomically defined and single-terminated surfaces by the typical chemical treatments and to induce interfacial redox-reactions in these nanoscopic transition metal oxides. To address this, we use the sacrificial layer exfoliation route, involving an all-perovskite epitaxial layer structure to fabricate freestanding $\hbox {SrTiO}_3$ membranes with high crystallinity and defined surface morphology. To study the interfacial redox-behavior of the singly $\hbox {TiO}_2$-terminated, annealed membrane, we employ the formation of oxygen vacancies in $\hbox {SrTiO}_3$, triggered by the low-pressure deposition of a thin $\hbox {LaAlO}_3$ layer epitaxially grown on the transferred $\hbox {SrTiO}_3$ layer. A mixed Ti$^{3+/4+}$ valence state is indicative of the induced transfer of oxygen ions from the confined $\hbox {SrTiO}_3$ membrane into the $\hbox {LaAlO}_3$ overlayer, resulting in an oxygen vacancy concentration of around $10^{21}~{\hbox {cm}^{-3}}$ in the confined $\hbox {SrTiO}_3$ membrane. Our results highlight that interfacial redox-reactions can be induced in $\hbox {SrTiO}_3$ membranes, which enables the ionic engineering of confined oxide heterointerfaces based on the freestanding oxide approach.

## Introduction

Transition metal oxides show a broad range of exciting functionalities, such as electronic, magnetic and ionotronic properties^1–4^. Perovskite oxides reflect a widely explored class of functional materials, which can be synthesized with atomically precise control of their properties. Particularly, epitaxy of perovskite thin films, heterostructures, and heterointerfaces by pulsed laser deposition (PLD) or molecular beam epitaxy has evolved as a major technique to tailor and combine perovskite oxides on the nanoscale^5,6^. This renders perovskite oxides as ideal model systems for electronic-ionic phenomena^7,8^. Available material combinations in oxide epitaxy, however, may be limited by structural constraints, such as space groups, lattice mismatch, or undesired cation interdiffusion under growth conditions^9–11^. In addition, the growth conditions applied in oxide epitaxy are typically not compatible with existing semiconductor technologies, severely limiting the projected application perspectives for oxide heterostructures.

These challenges might be overcome by the exfoliation and transfer of the deposited oxide layers, so called freestanding membranes, principally allowing to integrate oxide thin films with arbitrary host substrates or technological chip architectures. Such freestanding oxides are envisioned to yield material combinations not accessible by conventional epitaxy routes and, moreover, to potentially establish new physical phenomena that arise from restricted dimensions and confinement. For example, the electronic-ionic properties of such thin freestanding oxide layers may be expected to be fully dominated by space-charge-type defect distributions that form in the vicinity of their surfaces and interfaces^12–14^. This may imply for example an overall facilitated formation of defects or enhanced ion dynamics in the confined systems as compared to the bulk^15–17^.

Over the last years, different delamination strategies were employed for such freestanding oxides. Most popular are the chemical exfoliation routes involving the wet-etching of a sacrificial layer. Here, strontium aluminate (SAO) is a widely applied material as it easily can be dissolved in water at room temperature^18–20^, same solubility applies to SrO^21^. However, SAO departs from the ideal perovskite structure, lowering the epitaxial quality of possible perovskite oxide films grown on top, even though it can offer a low lattice mismatch to the chosen thin film via cation stoichiometry control^10,22^. In contrast, employing lanthanum strontium manganite (LSMO, $\hbox {La}_{0.7}$ $\hbox {Sr}_{0.3}$ $\hbox {MnO}_3$) as sacrificial layer, which can be etched by a HCl-based wet-chemical treatment, enables the growth of all-perovskite epitaxial heterostructures, offering additional potential for high crystallinity and structural integrity of the desired freestanding thin film^23–25^. Besides these sacrificial layer techniques, mechanical exfoliation routes through integration of weakly-bound two-dimensional (2D) material interlayers or spalling are currently applied for the fabrication of freestanding oxide membranes^26–28^. The integration of 2D materials in PLD processes, however, requires to apply inert gas atmospheres^29^, and the reported exfoliation yield is typically low compared to chemical exfoliation techniques^30^.

Among the perovskite family, strontium titanate (STO) is a model system for ionic-electronic phenomena, as its electronic properties are highly sensitive to its intrinsic defect structure and particularly to the formation of oxygen vacancies^31–33^. This makes STO an interesting material for oxide electronic applications^31^, memristive phenomena^34^, as well as energy applications^16,35,36^ and charge-transfer phenomena^15,31^. The investigation of charge transfer phenomena at interfaces in STO typically relies on a defined chemical termination. For instance, the dynamics of oxygen ion exchange may depend on the surface termination of STO^37^. At the same time, electronic and ionic charge transfer triggered by polar discontinuities at interfaces to STO, rely on a single termination of the STO as well^31,38,39^. Thus, the ability to generate a well-defined chemical termination and to study the redox-activity in freestanding STO membranes is a prerequisite to tailor ionic-electronic phenomena on the level of nanometer-sized transferable systems.

In this study, we employ the exfoliation and transfer of all-perovskite grown freestanding STO membranes. Subsequent to the transfer of the layers to $$\hbox {Al}_2$$$$\hbox {O}_3$$ single crystals, a single $$\hbox {TiO}_2$$-termination is achieved through a chemical and thermal treatment of the freestanding STO layer. The membrane is then utilized as a substrate layer for the atomically defined layer-by-layer growth of 5 unit cells (uc) of lanthanum aluminate (LAO) at low oxygen pressure, serving as a reducing agent for the confined membrane. We observe that a large density of oxygen vacancies can be generated within the STO membrane, naturally confining the redox-active volume to the thickness of the transferred membrane (here, 20 nm). Our results show that the control of the surface termination enables us to achieve a defined surface morphology and homogeneous step-terrace structure on the transferred oxide layer. A facile ion transfer from the membrane to the LAO overlayer is observed, allowing to tailor the ionic and electronic structure of the confined STO membrane.

## Results and discussion

**Figure 1:**
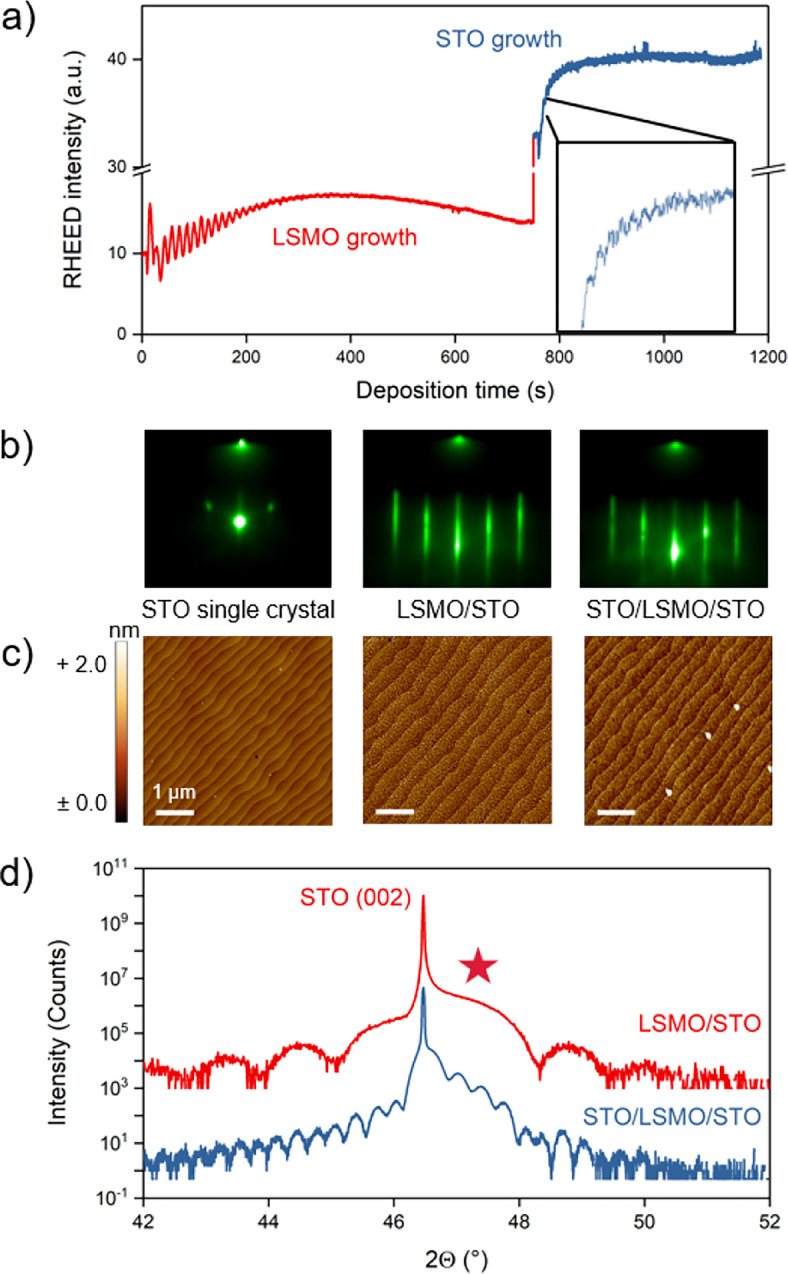
(**a**) RHEED oscillations recorded during PLD of 10 nm LSMO and subsequent 20 nm STO thin film layers. (**b**) RHEED patterns taken before deposition on the bare STO single crystal, after LSMO growth, and after STO growth (from left to right). (**c**) AFM scans of the bare STO single crystal, after LSMO growth, and after STO growth (from left to right, white bars scale with $1~\upmu$m). (**d**) XRD around the (002) peak of the sample after LSMO growth (top) and after STO/LSMO growth (bottom, shown spectra are offset along the y-axis for clarity). The LSMO (002) peak is marked by the red star.

Figure 1 shows the epitaxy of all-perovskite samples, involving the growth of a sacrificial LSMO interlayer and the subsequent deposition of STO. Fig. 1(a) displays the evolution of the reflection high-energy electron diffraction (RHEED) intensity during the growth, indicating a layer-by-layer growth mode for both, the LSMO and the subsequent growth of STO. For the LSMO sacrificial layer, a thickness of 10 nm was deposited, while for the desired freestanding oxide layer, 20 nm of STO were deposited subsequently.

Figure 1(b) shows the associated RHEED patterns received from the bare STO single crystal, after depositing the LSMO sacrificial layer and, finally, after the growth of the topmost STO layer. Starting with a classical 2D-diffraction pattern for STO single crystals, characterized by well-defined diffraction spots, a transition to a more streaky pattern is observed during the growth of LSMO, generally indicating that a two-dimensional, flat crystalline surface is maintained^40^. The proceeding evolution of the RHEED pattern during the growth of the additional STO layer towards a superimposed spot-like and streaky pattern further indicates a high crystallinity and smooth surface morphology, consistent with the observed layer-by-layer growth mode throughout the entire deposition process.

Figure 1(c) shows corresponding atomic force microscopy (AFM) scans of the different sample states, before growth, after LSMO deposition, and after STO growth (left to right). Prior to deposition, the original STO substrate was annealed, leading to the well-defined step terrace morphology with a root mean square (RMS) roughness of around 177 pm. After the deposition of 10 nm of LSMO, the surface shows a surface roughness of RMS $\approx 574~$pm, while the step terrace structure is conserved. A similar morphology is also adapted by the 20 nm thick STO layer, with a RMS surface roughness of 598 pm.

Figure 1(d) shows X-ray diffraction (XRD) data around the (002) peak of a 10 nm thick LSMO layer grown on an STO single crystal and of the same sample after growth of 20 nm STO on top. In both cases, the sharp peaks are attributed to the (002) reflection of the STO single crystal, whilst clear thickness fringes to both sides of the substrate peak stem from the thin films. For the LSMO grown on STO, a broadened shoulder next to the substrate peak is observed, which is assigned to the (002) LSMO film peak (marked by the red star symbol). After the growth of 20 nm STO on top, an additional modulation of the overall intensity is superimposed to the diffractogram due to the well-defined second overlayer. Furthermore, the STO thin film peak is overlapping in a well-aligned manner with the intense STO substrate peak, confirming the stoichiometric nucleation of the deposited material. These double-layer heterostructures serve as a well-defined starting point for the chemical delamination of the STO layer. Compared to the widely applied sacrificial layer approach involving SAO, LSMO and STO are both in perovskite structure, providing a structural continuity across the entire heterostructure^23^.

**Figure 2:**
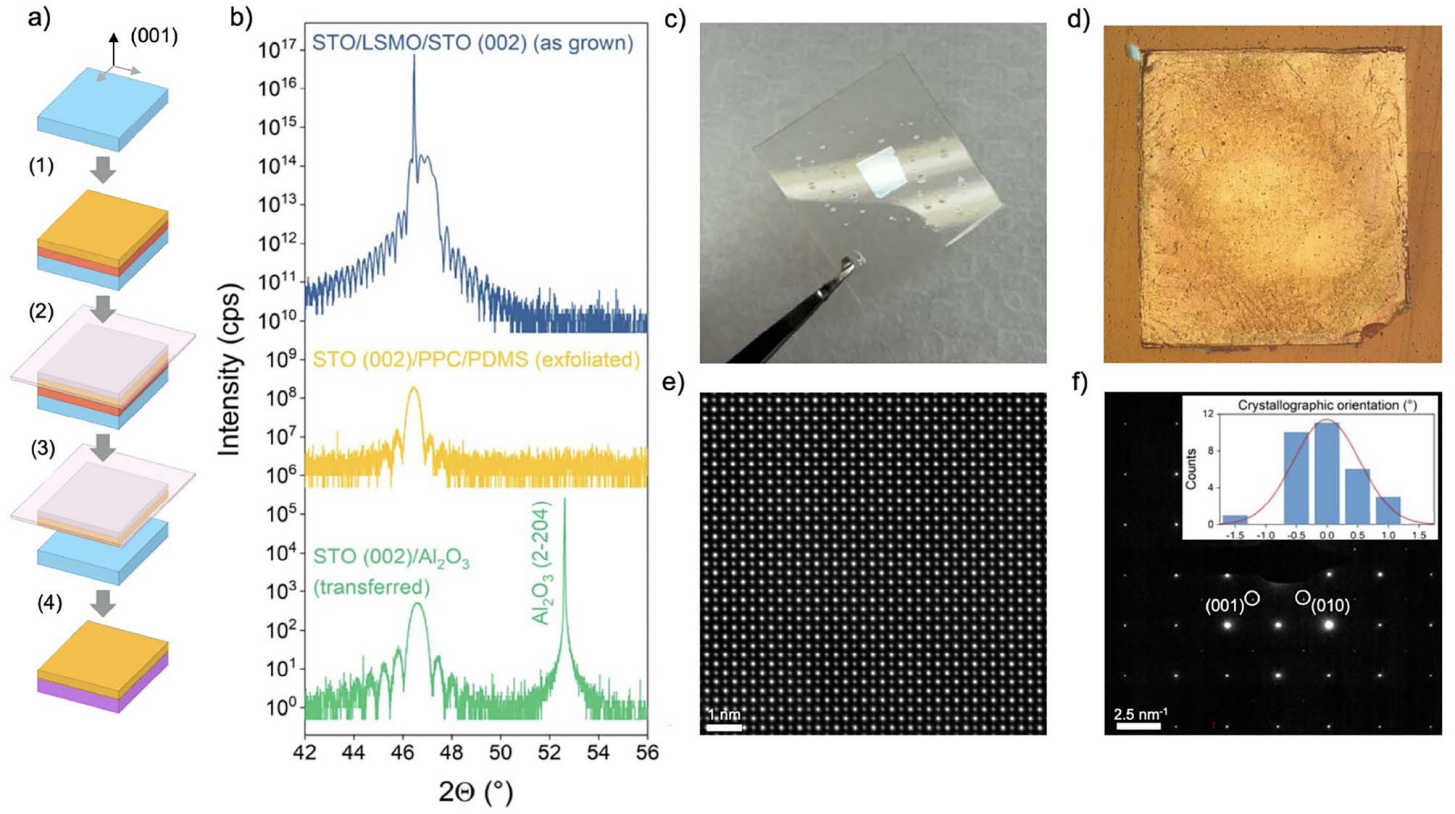
(**a**) Schematic of the exfoliation process, from sample fabrication and preparation, to exfoliation and transfer. (**b**) XRD around the (002) peak of an all-perovskite sample before exfoliation, of the remaining STO membrane attached to PDMS gel film, and of the STO membrane transferred to $\hbox {Al}_2$ $\hbox {O}_3$ host substrate (from top to bottom, shown spectra are offset along the y-axis for clarity). (**c**) Photography, and (**d**) light microscopy image of an exfoliated STO membrane attached to PDMS gel film. (**e**) HAADF-STEM image of an STO membrane on a silicon nitride membrane TEM grid, and (**f**) TEM diffraction pattern of the same sample with crystallographic orientation histogram as inset.

Figure 2(a) schematically shows the different steps for fabricating the freestanding membrane, using the sacrificial LSMO layer, and indicates the crystal orientation (001) of the samples. This already well-known exfoliation strategy involves the adequate pre-treatment of the substrate, followed by (1) the deposition of the LSMO layer and the desired functional STO layer. Subsequently (2), a polypropylene carbonate (PPC) layer is spin coated on top to stabilize and protect the freestanding membrane. Additionally, a polydimethylsiloxane (PDMS) handling layer is attached to the whole stack. To reach sufficient contact between the PPC and the PDMS, a uniform surface and low surface roughness of the PPC is required. Both can be controlled by the rotation during spin coating. Finally (3), the sample is dipped into the etching solution, containing HCl, KI and water, for six days to fully dissolve the LSMO. Afterwards (4), the remaining stack, i.e. the freestanding oxide layer attached to the polymer film and the handling layer, can be removed from the solution and rinsed in water (cf. Fig. 2 c). For the transfer of the freestanding STO to an epi-polished $$\hbox {Al}_2$$$$\hbox {O}_3$$ single crystal, the STO membrane is gently pushed onto the surface of the host substrate with a cotton swab. The sample is then placed on a hot plate (heated up to $$100$$ $$^{\circ }$$C) to decrease the adhesion between PPC and PDMS and, therefore, to easily remove the handling layer. The PPC is removed by dipping the remaining stack into acetone, finally delivering the freestanding layer on its new host substrate.

Figure 2(b) shows XRD data around the (002) peak of a freestanding STO sample in the different states of the transfer process. Compared to the as-grown sample (blue data set), the exfoliated sample (yellow) reveals the film peak in absence of the STO substrate peak as expected. We find clear thickness fringes for the freestanding STO layer attached to the PPC/PDMS stack. In the displayed $$2\Theta$$ range from 42 ° to 56 °, no signals of the PPC nor the PDMS are detected. Subsequent to the successful transfer of the freestanding STO layer to an $$\hbox {Al}_2$$$$\hbox {O}_3$$ single crystal, the (002) film peak and the thickness oscillations are still clearly visible as well as the substrate peak of $$\hbox {Al}_2$$$$\hbox {O}_3$$, indicating a widely coherent alignment of the crystal lattice of the transferred membrane with the underlying substrate. Note that an in-plane (twist) angle may be present between the host substrate and the transferred layer as it was controlled solely by a manual alignment of the membrane along the substrate edges^18,19,24^.

Fig. 2(c) shows a photography of an exfoliated $5 \times 5~{\hbox {mm}^2}$ STO membrane attached to the PPC/PDMS stack before the final transfer. Fig. 2(d) shows an optical microscopy image of the same sample after exfoliation and prior to transfer, indicating a high exfoliation yield. The optical image reveals an even surface on a millimeter length scale. However, crack formation becomes visible at larger magnification, indicating that the transferred layers are occasionally fractured on a length scale of 10 to $100~{\upmu \hbox {m}}$ (cf. Supplementary Fig. S1). As a result, macroscopic transport measurements such as in van der Pauw configuration are, at the current stage, challenging.

Figure 2(e) shows the corresponding high-angle annular dark field (HAADF)-scanning transmission electron microscopy (STEM) image after exfoliation. For this, a 20 nm thick STO membrane is transferred to a silicon nitride membrane TEM grid with holes etched through the silicon nitride membrane for direct imaging of the freestanding oxide layer. An optical microscopy image of the sample is shown in Supplementary Fig. S2a. The well-defined and ordered crystal structure confirms a coherent lattice structure that is evident in the entire investigated field of view. Fig. 2(f) shows the corresponding transmission electron microscopy (TEM) diffraction pattern of the same sample. The image reveals the typical cubic geometry of the perovskite structure of the freestanding STO, also evident in the electron diffraction pattern. Evaluating STEM images in more than 30 different positions, the relative variation of the in-plane crystallographic orientation was compared. The inset of Fig. 2(f) shows a histogram of the relative angular displacement of the crystal lattice (for further information see Supplementary Fig. S2b). The narrow distribution confirms the general lateral coherence of the lattice of the transferred STO membrane.

The transferred membrane on the inert $$\hbox {Al}_2$$$$\hbox {O}_3$$ host substrate can now be used to study the redox behavior of STO in a structurally well-defined and a vertically confined system. STO is particularly known for its interfacial charge transfer and redox behavior which can be triggered by ionic redox reactions as well as electronic charge transfer mechanisms^31,39,41^. Recently, it was reported that transferred freestanding oxides can be used as a substrate layer for the fabrication of oxide heterostructures^42,43^. Therefore, we employ the deposition of a sub-stoichiometric LAO thin film acting as a reducing agent to trigger a redox-response of the transferred STO layer.

**Figure 3:**
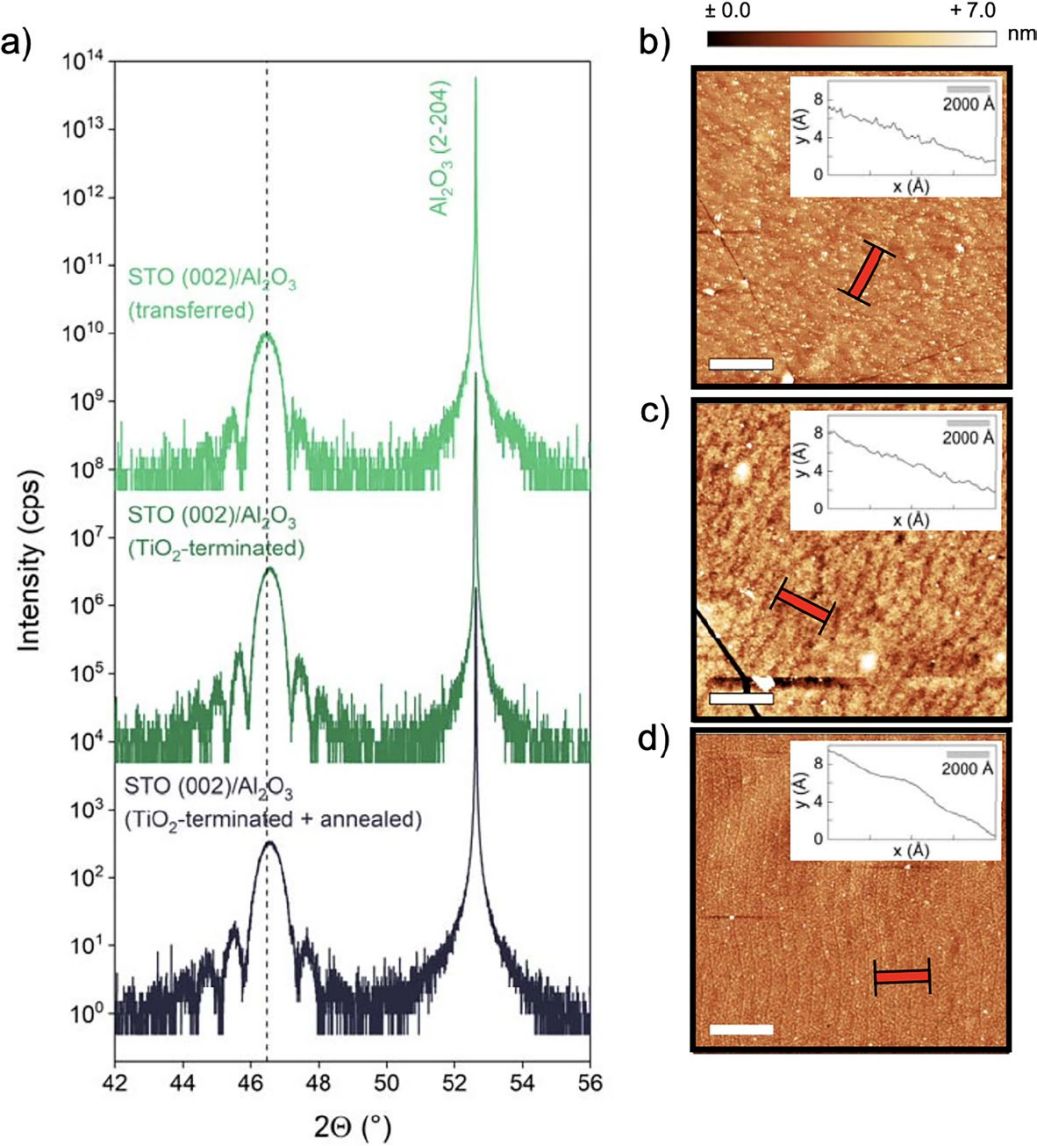
(**a**) XRD around the (002) peak of the STO membrane transferred to an $\hbox {Al}_2$ $\hbox {O}_3$ host substrate as-transferred, subsequent to $\hbox {TiO}_2$-termination by HF etching, and after final annealing (from top to bottom, shown spectra are offset along the y-axis for clarity). The dotted line marks the theoretical position of the thin film peak. AFM scans of the STO membrane (**b**) as-transferred, (**c**) after BHF treatment, and d) after final annealing (white bars scale with $1~\upmu$m). Red symbols show the orientation of the line profiles taken over $800~\text {nm}$ (shown as insets).

A common approach for achieving interfacial charge transfer to STO is to provide a defined chemical termination of the interface, which for single crystalline STO is achieved through a harsh wet-chemical treatment in buffered hydrofluoric acid (BHF)^44,45^. Here, prior to etching, the SrO at the surface is activated to form Sr(OH)$$_2$$ which is then dissolved in the acid. Adjusting the pH of the BHF and the etching time may significantly reduce the creation of etch pits, which are created especially at too low pH^46^, and, instead, leads to flat, well-terminated surfaces without remaining residues and without a significant reduction of the STO layer thickness. Fig. 3 shows the evolution of the crystal structure and the morphology of the transferred STO membrane under corresponding wet-chemical processing. Fig. 3(a) compares XRD data around the (002) peak of a freestanding STO layer transferred to an $$\hbox {Al}_2$$$$\hbox {O}_3$$ in its as-transferred state (top) to an STO membrane after etching in BHF for 2:30 min (center) and after an additional annealing step at $$950\,^{\circ }\text {C}$$ for 2 h in air subsequent to the BHF treatment (bottom). Referenced to the $$\hbox {Al}_2$$$$\hbox {O}_3$$ support peak, all STO thin film peaks are located very close to their expected position and depart less than +0.025 ° from the literature value of the STO (002) peak at 46.472 ° (cf. dashed line). This reflects a potential defect-induced lattice expansion within 0.2 pm, which is consistent with published research^47^. Compared to the literature^48–50^, this difference indicates a composition close to nominal stoichiometry. We further note that some peak positions may indicate a slight contraction of the STO unit cell (slight shift towards larger angles). This, however, may be an artefact of referencing the XRD measurement to the $$\hbox {Al}_2$$$$\hbox {O}_3$$ substrate peak, which may - due to the layer transfer and the individual miscut of the host substrate - not be in perfect alignment with the lattice of the transferred STO layer. In turn, rocking curves show a significant broadening of around $$\Delta \omega \approx 0.35~^\circ$$ (cf. Supplementary Fig. S3), which may indicate a larger degree of incoherence in the lattice structure of the STO layer. However, we note that after transfer, oxide membranes occasionally tend to possess wrinkles and cracks, which may cause additional broadening of the rocking curves, impeding direct evaluation of the lattice coherence.

Fig. 3(b) to (d) show the corresponding surface morphology of the STO membrane as-transferred (Fig. 3b), subsequent to the BHF treatment (Fig. 3c), and finally, after an annealing step in air (Fig. 3d). With this processing, the freestanding STO layer becomes increasingly smooth and forms atomic step terraces. Starting with a comparably high surface roughness of RMS $\approx 799~$pm, the chemical and thermal treatment leads to an RMS surface roughness of around 685 pm. A faint step terrace structure can be recognized already in the as-transferred membrane, indicating that this morphology is determined already during the initial PLD growth and maintained during the delamination process. Note that etch pits in the defined surface structure might occur due to the harsh chemical treatment of the sample by BHF. However, we observe a decreased surface roughness, indicating a continuous termination and a smooth layer surface is achieved. The line profiles shown as insets in Fig. 3(b–d) confirm the formation of single unit cell step heights. In this process, the BHF treatment primarily etches SrO entities from the surface of the membrane^45^. In comparison, the thermal treatment promotes a reorganization of the treated surface, providing defined atomic step terraces. We note, that a thermal treatment alone also results in a step terrace structure^19^, which, however, turns out less regular than for the BHF etched membrane (cf. Supplementary Fig. S4).

**Figure 4:**
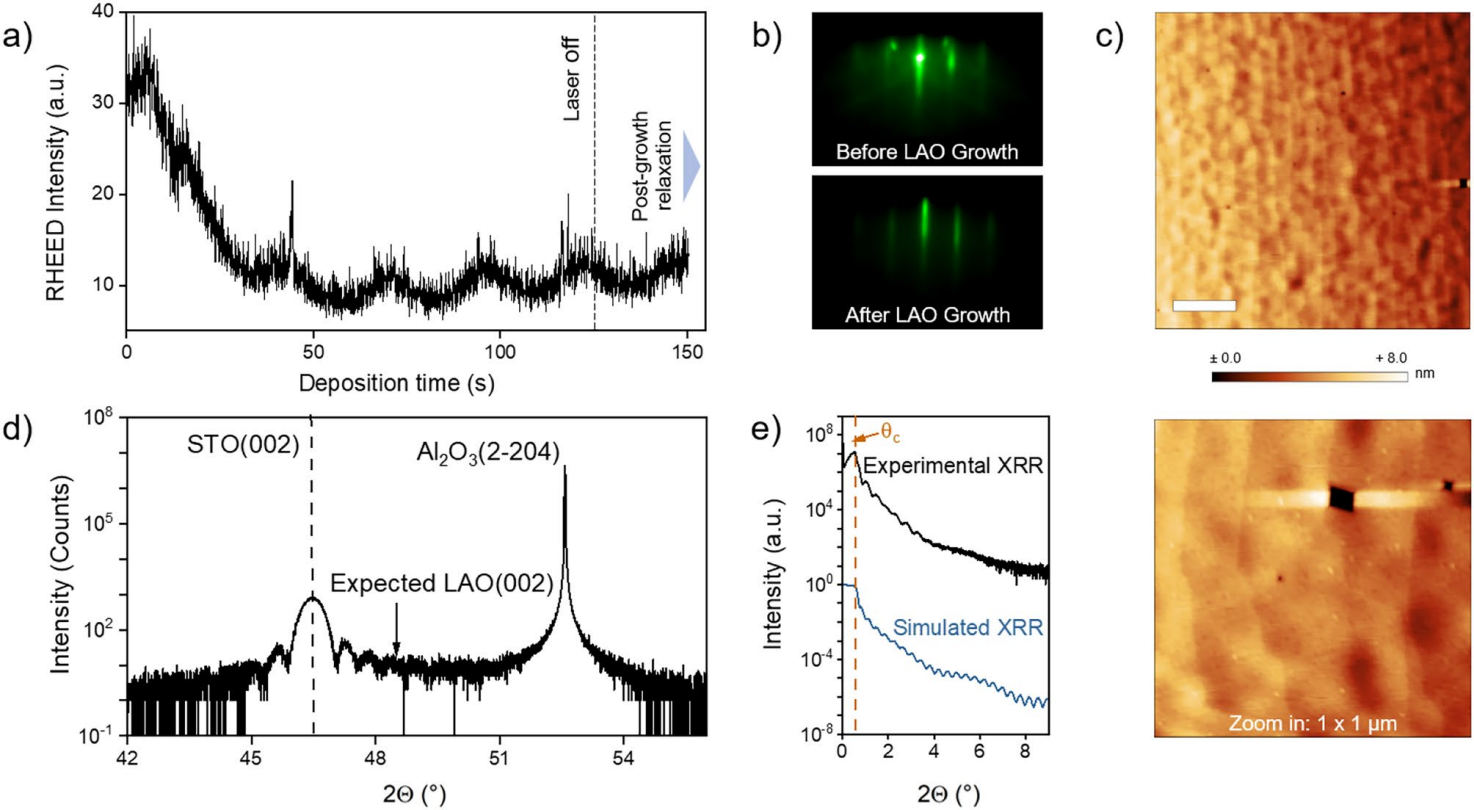
(**a**) RHEED oscillations recorded during PLD of 5 uc LAO on 20 nm thick, terminated and annealed STO layer transferred to an $\hbox {Al}_2$ $\hbox {O}_3$ host substrate. (**b**) RHEED pattern before (top) and after LAO growth (bottom). (**c**) AFM scans of the sample after LAO growth (white bar scales with $1~\upmu$m). (**d**) XRD around the (002) peak after LAO deposition. (**e**) XRR after LAO deposition experimental (top) and simulated (bottom).

After the termination step, we deposited LAO on top of the $\hbox {TiO}_2$-terminated STO layer. In order to specifically test the interfacial redox behavior of the membrane, the LAO layer was deposited at base pressure of $p_{\text {UHV}} = 1 \times 10^{-8}~\text {mbar}$, known to strongly trigger the formation of oxygen vacancies in STO substrates in standard epitaxy^51^. Fig. 4 a) shows the evolution of the RHEED intensity oscillations during the deposition, where every single maximum corresponds to one monolayer completely covering the substrate surface or the layer grown before, indicating that 5 uc of LAO are deposited in layer-by-layer growth mode, similar to the one observed for the epitaxy of LAO on conventional STO single crystals^8^. After stopping the deposition, an increase of the overall intensity is observed due to the post-growth relaxation of the sample. Fig. 4 b) shows the corresponding RHEED patterns before and after the growth of LAO, revealing a slightly streaky reflection pattern that is conserved during the deposition, whereas the overall intensity of the diffraction pattern decreases as frequently observed for the growth of LAO.

Fig. 4(c) shows the corresponding AFM scans of the sample after the LAO deposition. The step terrace structure is clearly visible for the STO and is also adapted by the thin LAO layer on top, indicating that both, the exfoliation and transfer of the STO membrane, its subsequent treatment as well as the LAO growth are achieved in a comparable manner as in standard epitaxy on $\hbox {TiO}_2$-terminated STO substrates.

Figure 4(d) shows XRD data around the (002) peak after growth of the 5 uc thick LAO layer. No clear thin film peak can be observed for LAO due to the low thickness of the layer and, hence, the low XRD intensity and peak broadening expected for the thin LAO layer. However, a faint additional intensity is observed around $$2\Theta \approx 48$$$$^\circ$$, corresponding to the expected position of the LAO (002) peak. The presence of the LAO layer becomes more evident in X-ray reflectivity (XRR) measurements as shown in Fig. 4(e), revealing a superimposed modulation of the XRR intensity expected for two layers with thicknesses of 20 nm (STO) and around 2 nm (LAO). The experimental result is in qualitative agreement with a corresponding simulation of the expected XRR intensity. Similar results for growth monitoring and X-ray measurements are found for an unterminated, only annealed sample (cf. Supplementary Fig. S5).

**Figure 5:**
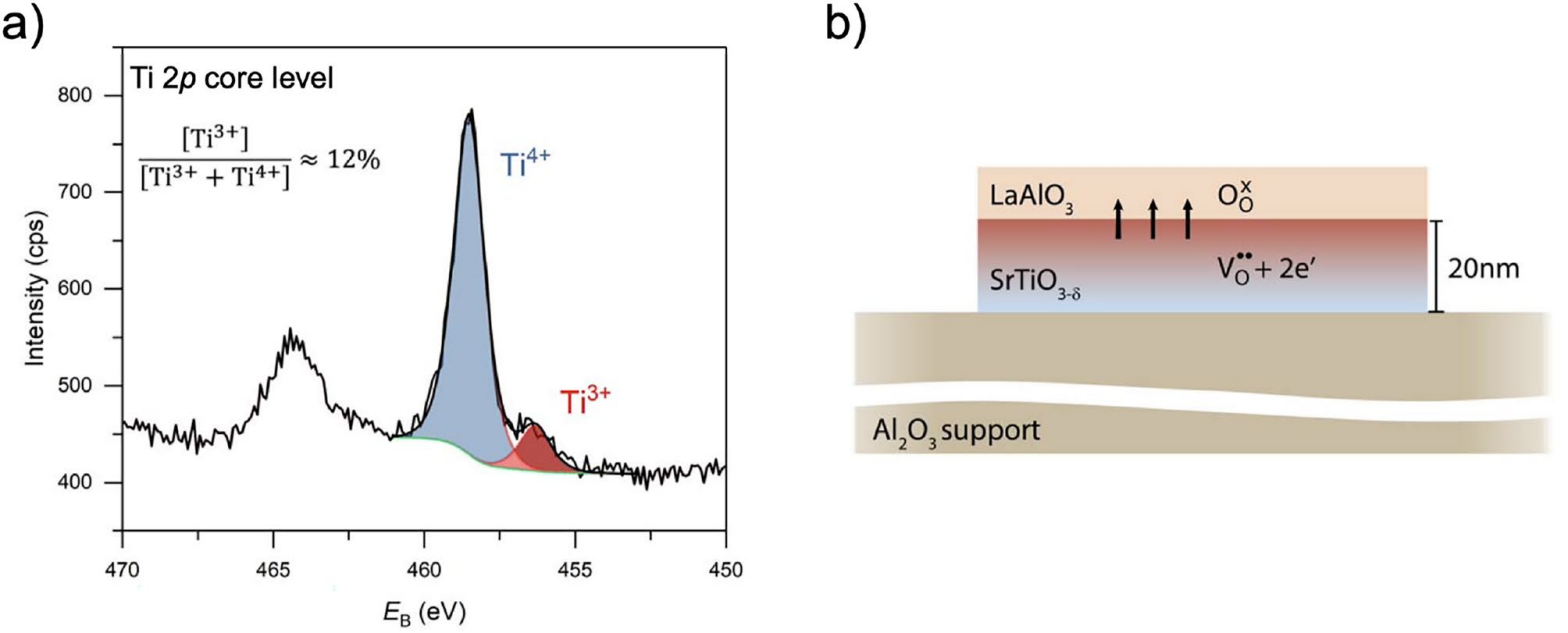
(**a**) XPS spectra around the Ti 2*p* core level for 5 uc LAO on a 20 nm thick STO layer transferred to an $\hbox {Al}_2$ $\hbox {O}_3$ host substrate. The STO layer was terminated and annealed prior to the LAO deposition. (**b**) Schematic of interfacial redox behavior between freestanding STO membrane and LAO layer.

In order to probe the redox-activity of the STO membrane, X-ray photoemission spectroscopy (XPS) of the Ti 2*p* core level was analyzed. Due to the low thickness of the LAO layer, the buried Ti 2*p* core level spectra can be recorded using Al ${\hbox {K}_\alpha }$ X-ray radiation with an information depth of several nanometers. Following the TPP2M formula^52^, the information depth of the XPS spectra can be estimated as $3 \cdot \lambda _{\text {IMFP}} \approx 6~$nm, with $\lambda _{\text {IMFP}}$ denoting the inelastic mean free path of electrons at a kinetic energy of 1000 eV such as in the case of the Ti core level spectra. We note, that the LAO overlayer will solely reduce the overall intensity of the XPS measurement. The resulting information depth within the STO layer is unaffected (corresponding O 1*s* core level spectra, reflecting mainly oxygen ions within the LAO overlayer, are displayed in Supplementary Fig. S6). As shown in Fig. 5 a), two clear contributions to the Ti 2*p* core level spectra can be identified and quantified, indicating a mixed Ti${^{3+}}$/Ti${^{4+}}$ valence state that is established after the deposition of the LAO layer. The clear shoulder towards lower binding energies indicates that a significant concentration of Ti${^{3+}}$ is present in the sample, exceeding the typical XPS detection limit. For comparison, a $1~\text {at}\%$-Nb-doped single crystalline reference sample reveals only negligible intensity in the same spectral region (cf. Supplementary Fig. S7), which is also confirmed for Nb-doped STO thin films in the same spectral region^53,54^.

Under the chosen reaction conditions at base pressure of $$p_{\text {UHV}} = 1 \times 10^{-8}~\text {mbar}$$, it is expected that the growing LAO layer getters oxygen ($${\hbox {O}_{\hbox {O}}^x}$$) from the neighboring STO, thereby creating oxygen vacancies ($${\hbox {V}_{\hbox {O}}^{\bullet \bullet }}$$) and electrons ($${\hbox {e}'}$$) in the STO membrane (cf. Fig. 5 b). Note that the deposition of LAO on $$\hbox {TiO}_2$$-terminated STO can also induce a 2-dimensional electron gas (2DEG) by electronic charge transfer mechanisms. However, we can estimate a contribution of around $$12~\%$$ of Ti$${^{3+}}$$ based on the area ratios of the components in the XPS spectra, which is a remarkably high value, and much higher than typically reported for the electronic-charge-transfer-based 2DEGs^39,55^. In fact, $$12~\%$$ of Ti$${^{3+}}$$ translate into a sheet density of around $$4\times 10^{15}~{\hbox {cm}^{-2}}$$, which exceeds the typical sheet densities observed for STO-based 2DEGs by about one order of magnitude^56–58^, indicating that the transfer of oxygen ions and a corresponding strong chemical reduction of the STO layer is responsible for the observed mixed valence state (cf. Fig. 5 b). Such interfacial redox-reactions were reported to occur also between $$\gamma$$-$$\hbox {Al}_2$$$$\hbox {O}_3$$ and STO, when $$\hbox {Al}_2$$$$\hbox {O}_3$$ is deposited at low growth pressure onto STO^59^. We note, that oxygen ions may hence also be exchanged between the STO membrane and the $$\hbox {Al}_2$$$$\hbox {O}_3$$ host substrate when kept at high temperature for several hours and further extended times. However, since the $$\hbox {Al}_2$$$$\hbox {O}_3$$ host substrate is already fully oxidized when the transfer of our STO membrane is applied, no immediate driving force for a reduction is expected to occur at the oxide layer/host substrate interface. We hence assume that the transfer of oxygen ions from the STO layer into the LAO film is required to induce the observed mixed valence state. This is also confirmed by a negligible Ti$${^{3+}}$$ contribution found for the as-transferred STO membrane (cf. Supplementary Fig. S7).

Within the XPS probing volume, we can furthermore estimate a characteristic Ti${^{3+}}$ volume concentration of the order of $2\times 10^{21}~{\hbox {cm}^{-3}}$, which corresponds to an expected oxygen vacancy concentration of $1\times 10^{21}~{\hbox {cm}^{-3}}$ (assuming charge neutrality via $[\text {Ti}^{+3}]~\hat{=}~ [\text {Ti}^{`}_x]= 2 [{{\hbox {V}}_{\hbox {O}}^{\bullet \bullet }}]$). This value is significantly higher than the typical concentrations obtained by thermodynamic reduction treatments, which are typically of order of $10^{18}$ to $10^{19}~{\hbox {cm}^{-3}}$^60^. Furthermore, it indicates that a significant amount of oxygen incorporated into the growing LAO layer stems from the buried STO membrane, rather than from the target or surrounding atmosphere, consistent with previous growth studies^61^. Moreover, the oxygen vacancies resulting from the redox process are confined to the 20 nm thick STO membrane and cannot spread into the bulk of the substrate such as reported for the standard epitaxy of LAO under these conditions. In that case, the resulting oxygen vacancies are typically distributed up to micrometers deep into the substrate^62^. Therefore, the local concentration of oxygen vacancies appears much higher than in conventional heterostructures and in fact compares to the local concentrations reported for heavily reduced filaments forming under resistive switching conditions^63^. The confinement of STO into a membrane-type system hence allows to concentrate an extremely high concentration of oxygen vacancies into a small volume, which may allow to study electronic correlations as well as defect-interactions based on intrinsically defect-doped systems.

Although a residual SrO termination is known to significantly reduce the kinetics of ion transport across the interface^31,37^, we note that we observe a comparable reduction of STO membrane that did not undergo BHF etching and, hence, reflect a mixed termination (cf. Supplementary Fig. S7). This indicates that under the chosen growth conditions, the chemical driving force for ion transfer and reduction of the STO membrane is strong enough to overcome the reduced kinetics of ion transport expected for SrO-terminated regions^64^. We also note, that the high concentration of Ti${^{3+}}$ is not caused by electron/ion neutralization applied during the XPS measurements. Instead, the main reason for the observed enhanced Ti${^{3+}}$ concentration might be the particular geometry of the membrane, impeding the redistribution of oxygen vacancies deep into the bulk^62^. Moreover, due to the finite size of the transferred layer, the effective reduction behavior may be determined by the surface reducibility of STO, which is reported to be facilitated compared to the bulk^12,13^, potentially adding a secondary aspect to the observed behavior. A rigorous thermodynamic analysis of the membrane properties will be required to deduce its effective reduction enthalpy.

## Conclusion

We demonstrate the all-perovskite sacrificial layer route involving LSMO to transfer STO membranes of high crystallinity and flat surface morphology, allowing the control of surface termination by chemical and thermal treatment. Utilizing the transferred STO membrane as a substrate layer, we achieve a layer-by-layer growth of 5 uc LAO at a base pressure of $$p_{\text {UHV}} = 1 \times 10^{-8}~\text {mbar}$$ via PLD. The high oxygen affinity of the LAO layer grown under such conditions reduces the buried STO layer, creating oxygen vacancies in the confined STO membrane and leading to a mixed Ti$${^{3+}}$$/Ti$${^{4+}}$$ valence state. The oxygen vacancy concentration of $$1\times 10^{21}~{\hbox {cm}^{-3}}$$, estimated based on the Ti$${^{3+}}$$ concentration, is significantly higher than the typical concentrations reached by a thermodynamic reduction. At the same time, the membrane dimensions yield a natural confinement for the oxygen vacancies, which dilute over micrometer length scales in standard epitaxy. Here, the use of an inert substrate with negligible oxygen ion mobility, such as $$\hbox {Al}_2$$$$\hbox {O}_3$$, acts as a diffusion barrier and effectively prevents oxygen ion transport from the substrate into the freestanding oxide layer. We note, that controlling the growth pressure during the deposition may be an additional control parameter to further confine redox-processes to the heterostructure interface, such as shown for $$\gamma$$-$$\hbox {Al}_2$$$$\hbox {O}_3$$/STO and amorphous-LAO/STO in the literature^59,65^. Our results show the particularity of such membrane-type systems, which are highly confined, allowing redox-reactions in size-limited and well defined nanoscopic transition metal oxides. This may open the door to novel studies on electronic correlations and defect-interactions in a number of intrinsically defect-doped materials.

## Experimental section

### Thin film synthesis

The epitaxial thin films are deposited by RHEED-controlled PLD (SURFACE systems + technology GmbH, Germany), operated with a KrF-excimer laser (Lambda Physik Lasertechnik, Germany) with a wavelength of $\lambda = 248~$nm. For the LSMO growth a laser fluence of $F = 1.70~\frac{\text {J}}{\text {cm}^2}$, a growth temperature of $T = 750\;^{\circ }\text {C}$, an oxygen partial pressure of $p_{\text {O}_2} = 0.24~\text {mbar}$, a laser frequency of $f = 5~\text {Hz}$ and a target-to-substrate distance of $d_{\text {T-S}} = 40~\text {mm}$ is used. Subsequent to the deposition, the sample is cooled down with a rate of $\frac{10\;^{\circ }\text {C}}{\text {min}}$ in the same background pressure. For the STO growth a laser fluence of $F = 1.05~\frac{\text {J}}{\text {cm}^2}$, a growth temperature of $T = 830~^{\circ }\text {C}$, an oxygen partial pressure of $p_{\text {O}2} = 0.10~\text {mbar}$ is used, whilst the other parameters stay the same. Prior to the thin film synthesis, epi-polished (001) STO substrates (Shinkosha Co. Ltd., Japan) are annealed at $950~^{\circ }\text {C}$ for 2 h in air to gain a precise step terrace structure. For the epitaxy of LAO, a laser fluence of $F = 0.96~\frac{\text {J}}{\text {cm}^2}$, a growth temperature of $T = 830~^{\circ }\text {C}$, a base pressure of $p_{\text {UHV}} = 1 \times 10^{-8}~\text {mbar}$, a laser frequency of $f = 1~\text {Hz}$ and a target-to-substrate distance of $d_{\text {T-S}} = 40~\text {mm}$ is used. After the deposition, the sample is annealed in the same background pressure and at the same temperature for 1 h, afterwards it is quenched to room temperature.

### Exfoliation and transfer

After sample fabrication, a layer of PPC is spin coated on top of the STO thin film. Beforehand, $$10~\text {g}$$ of PPC pellets are dissolved in $$100~\text {ml}$$ Anisole (Sigma-Aldrich/Merck KGaA, Germany). The PPC is spin coated with an acceleration of $$7500~\text {rpm}^2$$ to reach an even surface and subsequently baked at $$T = 100\;^{\circ }\text {C}$$ for $$10~\text {min}$$. Afterwards, the sample is attached to a 6.0 mil X4 PDMS gel film (Gel-Pak, USA). The whole sample stack is then placed into an etching solution, containing HCl ($$37~\%$$), KI (3M) and deionized $$\hbox {H}_2$$O with a ratio of 1 : 1 : 20. When the LSMO layer is fully dissolved, the STO substrate is removed and the PDMS gel film, still attached to the PPC/STO lamella, is isolated. The freestanding STO membrane is then pressed on the $$\hbox {Al}_2$$$$\hbox {O}_3$$ host substrate. By heating up the sample to $$T = 100\;^{\circ }\text {C}$$, the adhesion between PPC and PDMS decreases. This way, the gel film easily can be lifted off, while the oxide layer remains on the substrate. In a final step, the PPC is dissolved in acetone.

### Characterization and analysis

For the investigation of the surface morphology, a Cypher SPM (Research Asylum, Germany) atomic force microscope is used in tapping mode with silicon tips (NanoWorld AG, Switzerland). XRD is performed with a D8 ADVANCE diffractometer (Bruker AXS GmbH, Germany), operated with a monochromate Cu $\hbox {K}_{\alpha _{1}}$ radiation source in $2\Theta -\Theta$ geometry. HAADF-STEM imaging and TEM diffraction patterns are acquired on an aberration-corrected Thermo Fisher Scientic Spectra 300 STEM operated with an acceleration voltage of 200 kV. Diffraction patterns are acquired in TEM mode using a selected area aperture with a diameter of 650 nm. XPS measurements are conducted with a monochromate Omicron XM 1000 MkII Al $\hbox {K}_{\alpha }$ X-ray source and an Omicron EA 125 energy analyzer. A photoemission angle of 15 ° with respect to surface normal direction is used for all measurements and a pass energy of 23 eV is applied. Charge neutralization is conducted with an electron gun as well as an argon-ion gun. For fitting the data, the analysis software CasaXPS is used and the Ti 2$\hbox {p}_{3/2}$ core level is fitted by applying a Shirley background, for the peak shape a Gauss-Lorentz function with the ratio of 70 : 30 is applied. The Ti 2$\hbox {p}_{3/2}$ core level is fitted with two components representing the Ti$^{3+}$ and Ti$^{4+}$ signal and a FWHM constrain is applied from the Ti$^{4+}$ to the Ti$^{3+}$ with a distance of 2.2 eV.

## Data Availability

The datasets generated and analyzed during the present study are available from the corresponding authors on reasonable request.
